# Supplementary material for: Fifty years of evidence on perinatal experience among refugee and asylum-seeking women in Organization for Economic Co-operation and Development (OECD) countries: A scoping review
Source: PLoS One. 2023 Oct 26;18(10):e0287617. doi: 10.1371/journal.pone.0287617 (PMC10602334; doi:10.1371/journal.pone.0287617)
Supplement: Attachment — Submitted filename: Response to comments2.docx [file pone.0287617.s004.docx]

**Response to comments**

On behalf of the authors, I would like to thank the editor and all the reviewers for their comments and suggestions to improve the quality of the manuscript. We took all comments into consideration and addressed them within the manuscript. Below is a point-by-point response to the latest comments and the suggestions by the reviewer 1 including a description of the changes made to the older version of the manuscript.

| Comment | Response and description of changes / modifications |
| --- | --- |
| **Journal requirements:** | |
| Please review your reference list to ensure that it is complete and correct. If you have cited papers that have been retracted, please include the rationale for doing so in the manuscript text or remove these references and replace them with relevant current references. Any changes to the reference list should be mentioned in the rebuttal letter that accompanies your revised manuscript. If you need to cite a retracted article, indicate the article’s retracted status in the References list and also include a citation and full reference for the retraction notice. | Done |
| **Reviewer #1** | |
| Thank you for your response to feedback. I think the paper is much improved however, there are still some minor issues which need to be addressed. On page 5, line 84, you mention that no research has been done on asylum seekers and refugees in OECD countries. This strictly is not correct: See Heslehurst, N., Brown, H., Pemu, A., Coleman, H., & Rankin, J. (2018). Perinatal health outcomes and care among asylum seekers and refugees: a systematic review of systematic reviews. BMC medicine, 16(1), 1-25. which is an umbrella review across high income countries which includes OECD countries. There should be additional motivation or state what this study adds in addition to this existing body of research | We would like to thank the reviewer for his insightful comment and for drawing our attention to the paper by Heslehurst et al. (2018). We acknowledge that the cited paper included a general overview of some aspects of perinatal experience across refugees/asylum seekers in high income countries, which included some OECD countries. However, it did not deeply dive into the specific details of these experiences*.*  To be more accurate and address the points raised by the reviewer: we revised the two paragraphs at the end of background section to the following:  *Previous reviews have primarily focused on documenting adverse pregnancy outcomes among immigrant women, including refugees and asylum seekers (8,14–16). However, only a few studies have examined aspects related to their perinatal experience. For example, a comprehensive study by Heslehurst et al. (8) performed an umbrella review of perinatal health outcomes and care among refugees and asylum seekers, with some OECD countries represented in their sample. While the latter provided an insightful general overview of perinatal access and experiences among migrant women, the specific aspects of these experiences among refugees and asylum-seeking women in OECD countries are yet to be deeply explored.*  *Given the significant increase in humanitarian migration to OECD countries over the past decade, coupled with the organization’s commitment towards immigrants and refugees as part of the sustainable development goal 3 (17,18), it is imperative from a policy perspective to understand the various challenges faced by this vulnerable group for better integration, planning, and allocation of resources. Therefore, this scoping review aims to expand upon the existing literature by describing the general trends in perinatal health research among refugees/asylum seekers in OECD countries from 1970 to 2021 and synthesize the specific findings related to their perinatal experiences over the specified period.* |
| I also think that figure 3 should be removed as it is not clear how it contributes to the discussion. | The referenced figure was removed as recommended by the reviewer [Indicated by track changes] |
